# Refractory hypocalcaemia-induced laryngeal spasm requiring emergency intubation in a malnourished young woman: overcoming diagnostic and therapeutic barriers in resource-limited settings

**DOI:** 10.1186/s12245-026-01229-7

**Published:** 2026-04-15

**Authors:** Ayto A. Negash, Mehreteab T. Woudineh, Yitayew E. Mohammed, Eyob Z. Chafamo, Solomon Y. Tilahun, Petros T. Mekuria, Tekiy M. Bedore, Ashagre E. Hundeto

**Affiliations:** 1 Department of Emergency Medicine and Critical Care, Werabe Comprehensive Specialized Hospital, Werabe University, Werabe, Ethiopia; 2 Department of Emergency Medicine and Critical Care, Nigist Eleni Mohamed Memorial Comprehensive Specialized Hospital, Wachemo University, Hosanna, Ethiopia; 3 Department of Pediatric and Child Health, St. Paul’s Hospital Millennium Medical College, Addis Ababa, Ethiopia; 4 Department of Internal Medicine, Werabe Comprehensive Specialized Hospital, Werabe University, Werabe, Ethiopia; 5 Department of Neurosurgery, Werabe Comprehensive Specialized Hospital, Werabe University, Werabe, Ethiopia

**Keywords:** Hypocalcaemia, Laryngeal spasm, Upper airway obstruction, Vitamin D deficiency, Resource-limited setting, Emergency intubation, Cholecalciferol

## Abstract

**Background:**

Hypocalcaemia-induced laryngospasm is a rare but life-threatening cause of upper airway obstruction. Management is particularly challenging in resource-limited settings due to delayed laboratory results, limited monitoring, and unavailability of preferred medications.

**Case presentation:**

A 20-year-old previously healthy woman presented with severe stridor, respiratory distress, and carpopedal spasm. Laboratory findings (obtained after initial treatment) revealed ionized calcium 0.9 mmol/L (reference range: 1.12–1.32 mmol/L), magnesium 1.6 mg/dL (reference range: 1.7–2.3 mg/dL), and severe vitamin D deficiency (25-hydroxyvitamin D 11.83 ng/mL; severe deficiency < 10–12 ng/mL). Despite aggressive intravenous calcium gluconate boluses (2 g × 4 additional doses after initial), continuous infusion (up to 2 mg/kg/h), and magnesium replacement, laryngospasm progressed to complete airway obstruction with desaturation to 70%. Emergency rapid sequence intubation was performed with ketamine 100 mg (2.5 mg/kg) and vecuronium 4 mg. Because calcitriol was unavailable, intramuscular cholecalciferol 50,000 IU was administered as off-label loading. The patient was extubated after 72 hours and discharged after 8 days with normalized levels on continued cholecalciferol therapy.

**Conclusions:**

Favourable outcomes can be achieved in resource-limited emergency departments through clinically guided aggressive therapy, timely airway intervention, and pragmatic use of available long-acting vitamin D when preferred agents are inaccessible. This underscores the value of adaptable strategies in low-resource settings.

## Introduction

Hypocalcaemia is a potentially life-threatening metabolic disorder that increases neuromuscular excitability, manifesting as perioral numbness, carpopedal spasm, tetany, and, in severe cases, laryngospasm leading to acute upper airway obstruction and respiratory failure [1, 2]. Severe hypocalcaemia is defined as ionized calcium < 1.0 mmol/L (normal range approximately 1.12–1.32 mmol/L) whereas refractory hypocalcaemia is defined as persistent symptomatic hypocalcaemia despite adequate IV calcium and magnesium correction. Laryngospasm due to hypocalcaemia is rare in adults; most reported cases resolve with intravenous calcium alone, and presentations requiring emergency intubation are exceptionally uncommon [3–6]. Documented adult cases from resource-limited settings in sub-Saharan Africa are virtually absent, where diagnostic tools and active vitamin D analogues are frequently unavailable [7].

Standard management of severe symptomatic hypocalcaemia begins with an intravenous calcium bolus (1–2 g calcium gluconate or chloride over 5–10 min) followed by a continuous infusion (0.5–2 mg/kg/h), titrated to clinical response and serial levels, with close ECG monitoring to prevent arrhythmias [8]. Concurrent hypomagnesaemia must be corrected, as magnesium is a cofactor for parathyroid hormone (PTH) secretion and action; low magnesium impairs PTH release and causes end-organ resistance, rendering hypocalcaemia refractory until magnesium is repleted [6, 9, 10]. Vitamin D deficiency (severe when 25-hydroxyvitamin D < 10–12 ng/mL) frequently coexists and exacerbates the condition, particularly in malnourished patients. In resource-limited environments, ionized calcium assays, PTH measurement, and calcitriol are often unavailable, forcing reliance on clinical signs and locally available agents [9, 11, 12].

We report a case of refractory hypocalcaemia-induced laryngospasm causing upper airway obstruction, which required emergency intubation in a malnourished young woman at a resource-limited Ethiopian emergency department. This case demonstrates how timely airway management and pragmatic adaptation, using off-label intramuscular cholecalciferol when calcitriol was unavailable, enabled successful stabilization.

## Case presentation

A 20-year-old previously healthy Ethiopian female presented to the emergency department with a 1-day history of persistent, spontaneous muscle twitching in her hands and feet, associated with tingling sensations. She also experienced difficulty breathing and a high-pitched sound on inhalation with increasing shortness of breath. There was no prior history of thyroid, parathyroid, or renal disease, no medication use, and no family history of endocrine disorders.

On assessment, vital signs showed pulse rate 110 bpm, blood pressure 110/60 mmHg, respiratory rate 30–35 breaths per minute, and oxygen saturation 88% on room air. She appeared acutely ill and emaciated with marked suprasternal, intercostal, and subcostal retractions, severe continuous stridor, and carpopedal spasms. Mid-upper arm circumference (MUAC) was 18 cm, indicating severe malnutrition. Neck and chest examination revealed no goitre, lymphadenopathy, masses, or tenderness. Other systems were unremarkable.

Initial laboratory results (drawn after the first calcium bolus and 1 h of continuous infusion, received 6 h later from a private facility 200 km away) showed ionized calcium 0.9 mmol/L (normal 1.12–1.32 mmol/L), magnesium 1.6 mg/dL (normal 1.7–2.3 mg/dL), TSH 0.336 µIU/mL and repeated TSH 1.55 µIU/mL (normal 0.35–4.94 µIU/mL), total calcium 8.5 mg/dL (normal 8.4–10.2 mg/dL), and 25-hydroxyvitamin D 11.83 ng/mL (severe deficiency < 10–12 ng/mL). CBC, renal function, liver function, and other electrolytes were normal. Further workup (Phosphorus, PTH, free T3/T4, thyroid antibodies) was impossible due to financial constraints and unavailability. Ionized calcium testing was unavailable locally. Because frequent monitoring was not feasible, management was guided primarily by clinical signs (Table 1).

The only available ECG machine malfunctioned, so continuous cardiac monitoring was used as an alternative, which showed normal results. A bedside neck ultrasound was also performed and revealed normal findings.

Refractory hypocalcaemia was defined as persistent symptomatic hypocalcaemia (e.g., ongoing laryngospasm and respiratory distress) despite adequate intravenous calcium replacement and concurrent magnesium correction. Management was initiated at presentation (time 0) with a 2 g intravenous calcium gluconate bolus, followed by a continuous infusion at 1.5 mg/kg/h (patient weight 40 kg), and 3 L/min intranasal oxygen. Initially, there was transient improvement, but airway distress persisted. At 1 h, the stridor worsened, and the oxygen requirement increased to 4 L/min. In response, the calcium infusion rate was escalated to 2 mg/kg/h, and a 2 g magnesium sulfate bolus was administered. At 2 h, a third episode of airway distress occurred, prompting a second 2 g calcium gluconate bolus. By 4 h, there was further deterioration with increasing respiratory distress, leading to a third 2 g calcium gluconate bolus and a second 2 g magnesium sulfate bolus. At 6 h, a fifth episode of airway compromise, accompanied by worsening stridor, resulted in a fourth 2 g calcium gluconate bolus, while the calcium infusion rate was maintained at 2 mg/kg/h. By 8 h, critical deterioration occurred with SpO₂ dropping to 70% despite high-flow oxygen via a non-rebreather mask. Rapid sequence intubation was performed using ketamine 100 mg (2.5 mg/kg) and vecuronium 4 mg IV, with vecuronium chosen for its longer duration of action to prevent recurrent laryngospasm. Intubation was successful on the first attempt.

**Table 1:** Consolidated laboratory results

Parameter	Day 0	Day 4	Day 7	Day 14	Unit	Reference Range
White Blood Cell (WBC)	8	7	—	—	×10³/µL	2.90–20.00
Neutrophils	64	54	—	—	%	15–72
Haemoglobin (HGB)	14.3	13.6	—	—	g/dL	10–21
Haematocrit (HCT)	37	38.8	—	—	%	30–64
MCV	81	78	—	—	fL	71–125
Platelets (PLT)	136	146	—	—	×10³/µL	140–470
High-sensitivity Thyroid Stimulating Hormone (HsTSH)	**0.336**	1.55	—	—	µIU/mL	0.35–4.94 (lab) / 0.27–4.2 (standard adult)
Total Calcium	8.5	9.5	9.2	9.4	mg/dL	8.4–10.2 (lab) / 8.5–10.2 (standard)
Corrected calcium	9.116		—	—	mg/dL	8.4–10.2 (lab) / 8.5–10.2 (standard)
Ionized Calcium	**0.90**	1.12	1.14	1.21	mmol/L	1.12–1.32 (lab) / 1.05–1.3 (standard)
Magnesium	**1.6**	2.1	1.9	2.2	mg/dL	1.7–2.3
Sodium (Na⁺)	136	149	—	—	mmol/L	135–151
Potassium (K⁺)	4.4	3.8	—	—	mmol/L	3.5–5.1
Creatinine	0.52	—	—	—	mg/dL	0.0–1.3
Urea	21.8	—	—	—	mg/dL	12.8–42.8
Albumin	**3.23**–	—	—	—	g/dL	3.5–5.2
AST / ALT	31/0.37	—	—	—	U/L	0–41
**Vitamin D**	**11.83**	—	**24**	33	ng/mL	< 10–12 = severe deficiency; <20 = deficiency; 30 + = sufficient
**Urinalysis**	Normal	—	—	—	—	Negative

Note: Laboratory values on Day 0 were obtained after the initial calcium bolus and 1 hour of continuous infusion. RedBold values indicate abnormal results. Dashes (—) indicate test not repeated on that day

Immediately after intubation, 50,000 IU intramuscular cholecalciferol was administered as a loading dose (calcitriol unavailable). The patient was transferred to the ICU on mechanical ventilation with continued calcium infusion. She showed progressive improvement and was successfully extubated after 72 hours. She was discharged from the hospital after 8 days. At follow-up, ionized calcium and vitamin D levels had normalised. Cholecalciferol supplementation was initially given weekly and later changed to every 4 weeks for long-term maintenance.

## Discussion

Severe hypocalcaemia-induced laryngospasm remains a rare but life-threatening emergency in adults [3–5, 13]. Most reported cases resolve with intravenous calcium replacement alone, yet progression to complete upper airway obstruction requiring emergency intubation is exceptional, particularly in resource-limited settings where such presentations are rarely documented [7, 14].

In this patient, profound neuromuscular irritability from an ionized calcium level of 0.9 mmol/L was compounded by severe vitamin D deficiency (25-hydroxyvitamin D 11.83 ng/mL; severe deficiency defined as < 10–12 ng/mL) and concurrent hypomagnesaemia (1.6 mg/dL). Vitamin D deficiency is a common and important underlying cause of refractory hypocalcaemia, as it reduces intestinal calcium absorption and impairs PTH action, particularly in malnourished individuals [9, 10, 12, 15]. Hypomagnesaemia further exacerbates the refractoriness by impairing parathyroid hormone secretion and inducing end-organ resistance; magnesium repletion is therefore essential before calcium homeostasis can be restored [11, 16].

Management occurred in a resource-limited emergency department characterised by delayed laboratory results, unavailability of ionized calcium testing and calcitriol, and malfunction of the only ECG machine. Treatment relied on clinical response with aggressive calcium and magnesium replacement, followed by rapid sequence intubation when desaturation occurred despite high-flow oxygen. Off-label intramuscular cholecalciferol (50,000 IU loading dose) was used immediately after intubation because the preferred short-acting active vitamin D analogue was unavailable. This agent supported both acute stabilisation and long-term calcium homeostasis.

Vitamin D deficiency is highly prevalent in sub-Saharan Africa, including Ethiopia, where it affects a large proportion of the population due to malnutrition and socioeconomic factors [15]. In such settings, the combination of diagnostic constraints and limited access to essential medications makes management of severe metabolic emergencies particularly challenging [17]. Timely airway intervention served as a critical bridge, while pragmatic use of locally available medications compensated for the absence of ideal resources. Airway management in resource-limited emergency and ICU settings further requires rapid clinical decision-making when advanced monitoring is unavailable [18]. The successful extubation after 72 h and discharge after 8 days with normalised ionized calcium demonstrate that good patient outcomes remain possible through high clinical suspicion, response-guided therapy, and context-appropriate adaptations when guideline-recommended agents and recommended monitoring are inaccessible [19–22].

## Limitations

Limitations inherent to this resource-limited setting include delayed laboratory results (ionized calcium, magnesium, and vitamin D received 6 h later), unavailability of PTH and thyroid antibody testing, and malfunction of the only ECG machine, forcing reliance on clinical signs. These constraints reflect everyday realities in many low-resource emergency departments and highlight the value of pragmatic, patient-centred adaptations.

## Conclusion

In resource-limited emergency departments, refractory hypocalcaemia-induced laryngeal spasm can be successfully managed despite significant diagnostic, therapeutic, and monitoring limitations. Aggressive response-guided therapy combined with timely emergency intubation and pragmatic off-label use of long-acting cholecalciferol enabled full recovery. This case illustrates that good patient outcomes are possible in challenging low-resource settings through clinical judgment and adaptable use of locally available agents when ideal resources are absent. Such pragmatic approaches are critical for strengthening emergency care capacity and reducing mortality from treatable metabolic emergencies in low-resource settings.

## Data Availability

Available from corresponding author on reasonable request.

## Declarations

### Duplicate publication

This manuscript is original.

### AI statement

No AI tools were used in its preparation.

## References

[1] Shoback D. Clinical practice. Hypoparathyroidism. N Engl J Med. 2008;359(4):391–403. 10.1056/NEJMcp0803050. PubMed PMID: 18650515. 18650515 10.1056/NEJMcp0803050

[2] Cooper MS, Gittoes NJL. Diagnosis and management of hypocalcaemia. BMJ. 2008;336(7656):1298–302. 10.1136/bmj.39582.589433.BE. PubMed PMID: 18535072. 18535072 10.1136/bmj.39582.589433.BE PMC2413335

[3] Van Veelen MJ, Visser MF, Baggen MGA, Dees A. Hypocalcaemic laryngospasm in the emergency department. BMJ Case Rep. 2011;2011. 10.1136/bcr.11.2010.3555. PubMed PMID: 22707499. 10.1136/bcr.11.2010.3555 PMC3062878 22707499

[4] Joosen DAWA, Van De Laar RJJM, Koopmans RP, Stassen PM. Acute dyspnea caused by hypocalcemia-related laryngospasm. J Emerg Med. 2015;48(1):29–30. 10.1016/j.jemermed.2014.09.034. PubMed PMID: 25453856. 25453856 10.1016/j.jemermed.2014.09.034

[5] Melchers M, Van Zanten ARH. Management of hypocalcaemia in the critically ill. Curr Opin Crit Care. 2023;29(4):330–8. 10.1097/MCC.0000000000001059. PubMed PMID: 37395330. 37395330 10.1097/MCC.0000000000001059 PMC10328536

[6] Karunakaran P, Abraham D, Devadas G, Hussain Z, Kanakasabapathi R. The effect of hypomagnesemia on refractory hypocalcemia after total thyroidectomy: a single-center prospective cohort study. Indian J Endocrinol Metab. 2020;24(6):518–24. 10.4103/ijem.IJEM_681_20. PubMed PMID: 33643868. 10.4103/ijem.IJEM_681_20 PMC7906105 33643868

[7] Wakayo T, Belachew T, Vatanparast H, Whiting SJ, Vitamin D, Deficiency. Its predictors in a country with thirteen months of sunshine: the case of school children in central Ethiopia. PLoS ONE. 2015;10(3):e0120963. 10.1371/journal.pone.0120963. PubMed PMID: 25822900. 25822900 10.1371/journal.pone.0120963 PMC4387794

[8] Ryzen E, Wagers PW, Singer FR, Rude RK. Magnesium deficiency in a medical ICU population. Crit Care Med. 1985;13(1):19–21. 10.1097/00003246-198501000-00006. PubMed PMID: 3965244. 3965244 10.1097/00003246-198501000-00006

[9] Holick MF, Binkley NC, Bischoff-Ferrari HA, Gordon CM, Hanley DA, Heaney RP, et al. Evaluation, treatment, and prevention of vitamin D deficiency: an Endocrine Society clinical practice guideline. J Clin Endocrinol Metab. 2011;96(7):1911–30. 10.1210/jc.2011-0385. PubMed PMID: 21646368. 21646368 10.1210/jc.2011-0385

[10] Palacios C, Gonzalez L. Is vitamin D deficiency a major global public health problem? J Steroid Biochem Mol Biol. 2014;144(PART A):138–45. 10.1016/j.jsbmb.2013.11.003, PubMed PMID: 24239505. 24239505 10.1016/j.jsbmb.2013.11.003 PMC4018438

[11] Fuss M, Cogan E, Gillet C, Karmali R, Geurts J, Bergans A. Magnesium administration reverses the hypocalcaemia secondary to hypomagnesaemia despite low circulating levels of 25-hydroxyvitamin D and 1,25-dihydroxy vitamin D. Clin Endocrinol (Oxf). 1985;22(6):807–15. 10.1111/j.1365-2265.1985.tb00171.x. PubMed PMID: 3874724. 3874724 10.1111/j.1365-2265.1985.tb00171.x

[12] Calcium I of M (US) C to RDRI for VD and Ross AC, Taylor CL, Yaktine AL, Del Valle HB. Dietary reference intakes for calcium and vitamin D. Dri dietary reference intakes: calcium vitamin D. 2011;1–1116. 10.17226/13050. PubMed PMID: 21796828. 21796828

[13] Büyükcam F, Sönmez FT, Şahinli H. A delayed diagnosis: stridor secondary to hypocalcemia. Int J Emerg Med. 2010;3(4):461. 10.1007/s12245-010-0228-2. PubMed PMID: 21373324. 21373324 10.1007/s12245-010-0228-2 PMC3047863

[14] Chakrabarty A. Adult primary hypoparathyroidism: a rare presentation. Indian J Endocrinol Metab. 2013;17(Suppl 1):201. 10.4103/2230-8210.119571. PubMed PMID: 24251158. 10.4103/2230-8210.119571 PMC3830304 24251158

[15] Shaka MF, Hussen kabthymer R, Meshesha MD, Borde MT. Vitamin D deficiency among apparently healthy children and children with common medical illnesses in Sub-Saharan Africa: a systematic review and meta-analysis. Ann Med Surg (Lond). 2022;75. 10.1016/j.amsu.2022.103403. PubMed PMID: 35386789. 10.1016/j.amsu.2022.103403 PMC8977889 35386789

[16] Khan AA, Bilezikian JP, Brandi ML, Clarke BL, Gittoes NJ, Pasieka JL et al. Evaluation and management of hypoparathyroidism summary statement and guidelines from the second international workshop. J Bone Miner Res. 2022;37(12):2568–85. 10.1002/jbmr.4691. PubMed PMID: 36054621. 10.1002/jbmr.4691 36054621

[17] Fernandes C, Pereira L. Hypocalcemia in critical care settings, from its clinical relevance to its treatment: a narrative review. Anaesth Crit Care Pain Med. 2024;43(6). 10.1016/j.accpm.2024.101438. PubMed PMID: 39395659. 10.1016/j.accpm.2024.101438 39395659

[18] Sakane EN, Vieira MCC, Vieira GMM, Maeda SS. Treatment options in hypoparathyroidism. Arch Endocrinol Metab. 2022;66(5):651–7. 10.20945/2359-3997000000554. PubMed PMID: 36382754. 36382754 10.20945/2359-3997000000554 PMC10118816

[19] Di Maio S, Soliman AT, De Sanctis V, Kattamis C. Current treatment of hypoparathyroidism: theory versus reality waiting guidelines for children and adolescents. Acta Bio Medica: Atenei Parmensis. 2018;89(1):122. 10.23750/abm.v89i1.7118. PubMed PMID: 29633734. 29633734 10.23750/abm.v89i1.7118 PMC6357623

[20] Kataria S, Juneja D, Jain R, Veenith T, Nasa P. Airway management in the ICU and emergency department in resource-limited settings. Life. 2026;16(2). 10.3390/life16020195. 10.3390/life16020195 PMC12941945 41752833

[21] Hyder O, Wiener-Kronish JP, Shelton KT. Emergency intubation: a high-stakes procedure with critical gaps in understanding team impact. Am J Respir Crit Care Med. 2025;211(9):1725–6. 10.1164/rccm.202504-1043LE. PubMed PMID: 40540695. 40540695 10.1164/rccm.202504-1043LE PMC12432437

[22] Bulloch MN, Cardinale-King M, Cogle · Sarah, Radparvar S, Muhammad E, ·, Jagpal S, et al. Correction of electrolyte abnormalities in critically ill patients. Intensive Care Res 2024. 2024;4(1):1. 10.1007/s44231-023-00054-3.

